# One hundred and twenty kilometres of awareness: A pilot on psychological effects of a guided Camino in psychiatry residents

**DOI:** 10.1192/j.eurpsy.2026.11968

**Published:** 2026-07-31

**Authors:** M. Gerrickens, A. Oostindiër, B. Bus

**Affiliations:** 1 ETZ Hospital, Tilburg; 2 Aerrea, Wormer; 3 GGZ Oost Brabant, Boekel, Netherlands

## Abstract

**Introduction:**

Health care professionals tend to exhibit high senses of responsibility and self-sacrifice while ignoring stress symptoms. Consequently, burn-out and stress-related complaints are highly prevalent. Attention to health care professional wellbeing is increasing. Measures aimed at reducing stress are frequently focussed on time management and organisational factors. Interventions on intrapersonal traits during early-stage career might prove effective.

**Objectives:**

To evaluate the effect of a guided ‘Camino de Santiago’ pilgrimage on stress, interoceptive awareness and psychological schemes in psychiatry residents.

**Methods:**

An interventional study was performed in psychiatry residents from GGZ Oost Brabant. Participants went on a week-long pilgrimage guided by an external health care organization. Each day started with mindfulness, meditation or experience-based exercises. Hikers were encouraged to experience their surroundings and tendencies walking alone in silence for optimal introspection. After lunch, socializing was allowed. In the evening, experiences were shared in the group or subgroups. Residents maintaining their usual working activities served as controls. Three questionnaires were completed before and after Camino: Copenhagen Burnout Inventory for stress, Multidimensional Assessment of Interoceptive Awareness for bodily experiences and Young Schema Questionnaire 3 for psychological schemes. The top-5 prevalent schemes were studied.

**Results:**

Thirteen residents participated (9 women, mean age 32 (27-47) years). Demographics of hikers (n=8) and controls (n=5) were similar. Stress-levels remained unaltered. Several domains of interoceptive awareness improved in hikers: attention regulation (0.9 [0.5-1.5], p=0.02), emotional awareness (0.5 [0.1-1.2], p=0.03), self-regulation (0.5 [0.1-0.9], p=0.03), listening to the body (0.8 [0.1-1], p=0.05). Controls showed no changes. The top-5 prevalent schemes were unrelenting standards (n=12), self-sacrifice (n=10), approval seeking (n=7), punitiveness (n=6), emotional inhibition and social isolation (both n=5). Hikers showed decreases in unrelenting standards (-0.3 [-0.9-0], p=0.04), approval seeking (-0.6 [-0.8-0], p=0.03), punitiveness (-0.3 [-0.9- -0.1], p=0.04) and social isolation (-0.4 [-1.1- -0.1], p=0.03). In controls, approval seeking decreased (-0.4 [-1.1- -0.3], p=0.03).

**Conclusions:**

A week-long guided hike with experience-based interventions did not alter self-reported stress on the short term in psychiatry residents. Interestingly, several domains of interoceptive awareness and highly prevalent schemes, putting people at risk for mental health problems, improved in the hikers. Future studies could focus on qualitative results and longer-term effects.

**Disclosure of Interest:**

None Declared

